# BMCMDA: a novel model for predicting human microbe-disease associations via binary matrix completion

**DOI:** 10.1186/s12859-018-2274-3

**Published:** 2018-08-13

**Authors:** Jian-Yu Shi, Hua Huang, Yan-Ning Zhang, Jiang-Bo Cao, Siu-Ming Yiu

**Affiliations:** 1 School of Life Sciences, Northwestern Polytechnical University, Xi’an, 70072 China; 2 School of Software and Microelectronics, Northwestern Polytechnical University, Xi’an, 70072 China; 3 School of Computer Science, Northwestern Polytechnical University, Xi’an, 70072 China; 4 Department of Computer Science, The University of Hong Kong, Hong Kong, 999077 China

**Keywords:** Microbe-disease association, Matrix completion, Prediction, Machine learning

## Abstract

**Background:**

Human Microbiome Project reveals the significant mutualistic influence between human body and microbes living in it. Such an influence lead to an interesting phenomenon that many noninfectious diseases are closely associated with diverse microbes. However, the identification of microbe-noninfectious disease associations (MDAs) is still a challenging task, because of both the high cost and the limitation of microbe cultivation. Thus, there is a need to develop fast approaches to screen potential MDAs. The growing number of validated MDAs enables us to meet the demand in a new insight. Computational approaches, especially machine learning, are promising to predict MDA candidates rapidly among a large number of microbe-disease pairs with the advantage of no limitation on microbe cultivation. Nevertheless, a few computational efforts at predicting MDAs are made so far.

**Results:**

In this paper, grouping a set of MDAs into a binary MDA matrix, we propose a novel predictive approach (BMCMDA) based on Binary Matrix Completion to predict potential MDAs. The proposed BMCMDA assumes that the incomplete observed MDA matrix is the summation of a latent parameterizing matrix and a noising matrix. It also assumes that the independently occurring subscripts of observed entries in the MDA matrix follows a binomial model. Adopting a standard mean-zero Gaussian distribution for the nosing matrix, we model the relationship between the parameterizing matrix and the MDA matrix under the observed microbe-disease pairs as a probit regression. With the recovered parameterizing matrix, BMCMDA deduces how likely a microbe would be associated with a particular disease. In the experiment under leave-one-out cross-validation, it exhibits the inspiring performance (AUC = 0.906, AUPR =0.526) and demonstrates its superiority by ~ 7% and ~ 5% improvements in terms of AUC and AUPR respectively in the comparison with the pioneering approach KATZHMDA.

**Conclusions:**

Our BMCMDA provides an effective approach for predicting MDAs and can be also extended to other similar predicting tasks of binary relationship (e.g. protein-protein interaction, drug-target interaction).

## Background

Human intestine provides a nutrient-rich and temperature-constant habitat for microbes, such that the microbes have a mutualistic association with their host [1]. Diverse communities of microbes, especially bacteria, are found by sequencing techniques (e.g. 16S ribosomal RNA sequencing) in human bodies [2]. It is surprising that the number of genes in human microbiome is up to 5 million [3]. Both these genes and their products are participating in a diverse range of biological activities, such as metabolic capabilities, pathogens, immune system, and gastrointestinal development [4]. It can be said that they somehow serve as a physiological complement in the human body. Meanwhile, both communities and populations of microbes can be significantly influenced by their dynamic habitat in the human body. Diverse environmental variables, such as season [5], host diet [6], smoking [7], hygiene [3] and use of antibiotics [8], may change the habitat of microbes frequently. This kind of mutualistic associations between human host and its microbiota would cause the modifications of transcriptomic, proteomic and metabolic profiles in the human body. However, some of the modifications could be harmful.

Beyond the fact that microbe is the main player in the pathogenic mechanism of infectious diseases, an increasing number of clinical studies have demonstrated that the microbiota in human body is strongly associated with a wide range of human non-infectious diseases, such as cancer [9], obesity [10, 11], diabetes [12, 13], kidney stones [14] and systemic inflammatory response syndrome [15]. Nevertheless, people have only a limited understanding of what microbes cause the diseases and how they do.

Fortunately, the increasing number of experimentally validated associations between human non-infectious diseases and microbes enable us to perform a systematic analysis on microbe-disease associations (MDAs). For example, Ma et al. recently published the first database of MDA, Human Microbe-Disease Association Database (HMDAD), by collecting a large number of MDAs from previously published literature [16]. The MDA entries in HMDAD mainly focuses on experimentally supported associations between diverse microbes and non-infectious diseases, and all of them are experimentally supported with sufficient samples. The systematic analysis on a large scale of MDAs provides a new insight to discover the mechanism of microbe-related non-infectious diseases [17]. As one of the most important steps towards that goal, the identification of MDA is helpful to understand how non-infectious diseases develop and exploit novel methods for disease diagnosis and therapy. However, traditional experiment-based approaches for discovering MDAs are time-consuming and costly. Even worse, many bacteria cannot be cultivated at all by current culturing bio-techniques [18].

As the complement of biological experiment-based approaches, computational approaches are promising to rapidly screen MDA candidates, such that the further biological validation reduces the cost and time significantly. More importantly, they are expected to output the MDA candidates involving uncultivable microbes. A few efforts have been made to develop computational models for the large-scale MDA prediction. Recently, a pioneering work developed an approach, KATZHMDA, for predicting potential MDAs on a large scale [19]. After constructing an MDA network based on HMDAD, KATZHMDA models MDA prediction as link prediction on the network.

**Fig. 1:**
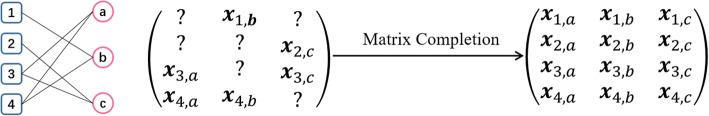
A Toy MDA Example for Matrix Completion. The left matrix is an observed matrix, in which *x*_*ij*_ are the observed MDA entries and ‘?‘s denote the unobserved microbe-disease pairs. The right matrix is the expected matrix with fully observed entries

In this work, by modeling MDA prediction as a problem of matrix completion (Fig. 1), we propose a new predictive approach based on Binary Matrix Completion (BMCMDA) to predict potential MDAs on a large scale by only using a set of approved microbe-disease associations. The following sections are organized as follows. Section Method first introduces the basic idea to model MDA prediction, then represents the algorithm of binary matrix completion. Section Experiments briefly describes the benchmark dataset of MDA, shows how to tune the parameters in the proposed model, and demonstrates the ability of BMCMDA by the comparison with other state-of-the-art approaches. The final section draws our conclusion. In addition, human non-infectious diseases are termed as ‘diseases’ and their microbes in the body are termed as ‘microbes’ in the following texts for concision.

## Methods

### Problem formulation

Given *p* kinds of microbes *M* = {*m*_*i*_}, *q* types of diseases *D* = {*d*_*j*_}, and a set of associations between them, we aim to deduce or predict new potential associations among them. Those microbe-disease associations can be organized into a *p* × *q* binary adjacent matrix **A** = {*a*_*ij*_}, where *a*_*ij*_ =  + 1 and *a*_*ij*_ =  − 1 account for whether *m*_*i*_ is associated with *d*_*j*_ or not respectively, and *a*_*ij*_ = ? if the association between *m*_*i*_ and *d*_*j*_ is NOT observed. Our problem is to deduce how likely those unobserved entries are MDAs (Fig. 1).

Matrix completion is one of the popular techniques to deduce the relationship between two types of objects (i.e. users and items) in recommendation system. However, the standard algorithms of matrix completion working on real-valued or categorical observations fail to infer the binary relationship between the objects [20], such as MDA prediction. Therefore, we adopted a different technique in the next section.

### Binary matrix completion

We state the problem as a matrix completion with 1-bit observation, in which each observed entry represents a positive (yes) or negative (no) response to MDA. Such a binary matrix completion can be defined as a generalized linear model,

1 $$ {a}_{ij}=\left\{\begin{array}{cc}+1& {x}_{ij}+{z}_{ij}\ge 0\\ {}-1& {x}_{ij}+{z}_{ij}<0\end{array}\right. $$

where only a subset Ω of entries of **A** is observed, **X** = {*x*_*ij*_} is a low-rank parameterizing distribution matrix of **A**, and **Z** = {*z*_*ij*_} is a stochastic matrix containing noise. The recovery of matrix **X** is usually transformed to another form to solve as follows [21].

**Fig. 2:**
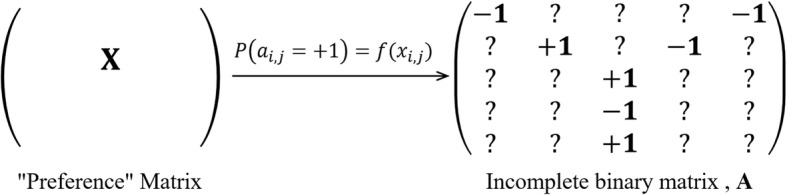
Binary matrix completion. The left matrix is the latent preference matrix. The right matrix is the observed matrix, in which the observed entries are labelled with ‘+ 1’ if an MDA is found, with ‘-1’ if a non-MDA is found, and ‘?’ if the entry is not observed

Given an incomplete observed MDA matrix **A** ∈ ℝ^*p* × *q*^, a subset of its observed entry subscripts Ω ⊂ [*p*] × [*q*] and a differentiable function *f* : ℝ → [0, 1], we observe

2 $$ {a}_{ij}=\left\{\begin{array}{cc}+1& \mathrm{with}\ \mathrm{the}\ \mathrm{probability}\kern0.5em f\left({x}_{ij}\right)\\ {}-1& \mathrm{with}\ \mathrm{the}\ \mathrm{probability}\kern0.5em 1-f\left({x}_{ij}\right)\end{array}\right.\kern1em \mathrm{for}\forall \left(i,j\right)\in \Omega $$

where [d] denotes the set of integers {1,..,d}. In other words, the entries of **A** depend on a *p* × *q* underlying low-rank preference matrix **X** = {*x*_*ij*_} ∈ ℝ^*p* × *q*^ somehow (Fig. 2).

We assume that the subscript subset Ω follows a binomial model, in which the subscript (*i*, *j*) ∈ [*p*] × [*q*] of each observed entry in A occurs with probability m/(pq) independently, where m is the cardinality (the number of observed entries) of Ω. The assumption reflects p×q independent experiments, of which each determines microbe-disease associations with m/(pq) success probability.

In addition, if we suppose that the entries of the underlying noising matrix **Z** are independently and identically drawn from the distribution, whose cumulative distribution function (CDF) is given by *F*_*Z*_(*x*) = *P*(*z* ≤ *x*) = 1 − *f*(−*x*), then the model in Formula (2) reduces to its special case in Formula (1). In such a sense, the selection of CDF *f* is equivalent to that of Z. Thus, X can be also viewed as a parameter of a distribution.

Since our aim is to determine the likelihood that a microbe would be associated with a particular disease, we naturally model MDA prediction as the problem that recovers the latent low-rank matrix **X**.

When defining the CDF *f*(*x*_*ij*_) = 1 − Φ(−*x*_*ij*_/*σ*) = Φ(*x*_*ij*_/*σ*), where Φ is the cumulative distribution function of a standard Gaussian (a standard mean-zero Gaussian with variance σ^2^ for the noising matrix **Z**), Formula (2) captures a probit regression model. Thus, the recovery of **X** can be achieved by solving the following optimization problem [21],

3 $$ {\displaystyle \begin{array}{l}\widehat{\mathbf{X}}=\underset{\mathbf{X}}{\arg \max }{F}_{\Omega, \mathbf{A}}\left(\mathbf{X}\right)\\ {}{F}_{\Omega, \mathbf{A}}\left(\mathbf{X}\right)=\sum \limits_{\left(i,j\right)\in \Omega}\left(B\left({a}_{ij}=+1\right)\log \left(f\left({x}_{ij}\right)\right)+B\left({a}_{ij}=-1\right)\log \left(1-f\left({x}_{ij}\right)\right)\right)\\ {}f\left({x}_{ij}\right)=1-\Phi \left(-{x}_{ij}/\sigma \right)=\Phi \left({x}_{ij}/\sigma \right)\\ {}s.t.\kern1em {\left\Vert \mathbf{X}\right\Vert}_{\ast}\le \sqrt{rpq}\end{array}} $$

where *B*(*ε*) is the binary indicator function for an event *ε*(i.e. *B*(*ε*) = 1 if *ε* occurs and 0 otherwise), Φ(*x*_*ij*_/*σ*) ∈ ℝ → [0, 1] is the cumulative distribution function of a standard Gaussian distribution with variance *σ*^2^, and *r* is the expected rank of **X**.

Consider that Formula (3) is just a special instance of the general formulation

4 $$ \underset{\mathbf{x}}{\min}\kern1em f\left(\mathbf{x}\right)\kern1em \mathrm{subject}\ \mathrm{to}\kern1.25em \mathbf{x}\in \boldsymbol{C} $$

where *f*(**x**) is a smooth convex function from ℝ^*n*^ → ℝ, and ***C*** is a closed convex set in ℝ^*n*^. In particular, defining ***V*** as the bijective linear mapping that vectorizes ℝ^*p* × *q*^ to ℝ^*pq*^, we have *f*(**x**) =  − *F*_Ω, **A**_(***V***^−1^**x**) and ***C = V***({**X** : ‖**X**‖_∗_ ≤ *τ*}). Therefore, non-monotone Spectral Projected Gradient (SPG) can be applied to solve the above optimization [22]. It is an iterative algorithm, which requires at each iteration the evaluation of *f*(**x**), its gradient *g*(**x**) = *∇f*(**x**) and an orthogonal projection ***P***_***C***_(**v**) onto ***C***, ***P***_***C***_(**v**) = arg min ‖**x** − **v**‖_2_ subject to  **x** ∈ ***C***. Since the orthogonal projection onto the nuclear-norm ball ***C*** amounts to singular-value soft thresholding [23], the projection is equivalent to

5 $$ {\boldsymbol{P}}_{\boldsymbol{C}}\left(\mathbf{X}\right)={\boldsymbol{S}}_{\lambda}\left(\mathbf{X}\right):= \mathbf{U}\max \left\{\boldsymbol{\Sigma} -\lambda \mathbf{I},0\right\}{\mathbf{V}}^T $$

where $\mathbf{X}\overset{SVD}{=}{\mathbf{U}\boldsymbol{\Sigma } \mathbf{V}}^T$,**Σ** =  *diag* (*σ*_1_, …, *σ*_*n*_), the maximum operation is taken entry-wise and *λ* ≥ 0 is the smallest value for which ${\sum}_{i=1}^n\max \left\{{\sigma}_i-\lambda \right\}\le \tau$.

### Cross validation

As a standard technique, cross-validation (CV) is popularly adopted to evaluate the performance of machine learning models and estimate their power of generalization on future samples. Usually, there are two kinds of CV, k-fold cross-validation (k-CV) and leave-one-out cross-validation (LOOCV).

In the scheme of k-CV, all the observed samples are randomly split into k subsets of approximately equal size. Among them, one subset is taken as the testing set, in which the samples are masked as unobserved. Meanwhile, the remaining k-1 subsets are merged as the training set, in which the observed samples are used to train a predicting model. Once the training is done, the predicting model is performed on the testing set and outputs the confidence scores of being observed samples for all the masked samples. This procedure repeats k times by taking each subset as the testing set in turn. In each round of k-CV, the performance of the predicting model is measured and recorded. Its final performance is defined as the average of the performance in all the rounds.

LOOCV can be regarded as an extreme case of k-CV, where k is equal to the number of observed samples. In each step of LOOCV, each observed sample is blinded as an unobserved one and the remaining observed samples are used to build the predicting model. The procedure of LOOCV takes each of the observed samples as the testing sample in turn. When the number of samples is enough large, the results of k-CV and LOOCV have no significant difference in statistics.

The performance of MDA prediction is measured by Receiver Operating Characteristic (ROC) curve as well as Precision-Recall (PR) curve. Two measuring metrics adopted are both the Area Under ROC curve (AUC) and the Area Under PR curve (AUPR). One could easily obtain other metrics, such as true positive rate (TPR, Recall, or Sensitivity) and false positive rate (FPR, 1-Specificity), by setting thresholds on ROC or PR curves.

## Results and discussion

### Dataset

We adopted the same dataset of MDAs as that in [19]. The dataset was originally collected from the Human Microbe-Disease Association Database (HMDAD, http://www.cuilab.cn/hmdad), which was built in 2016 and published in 2017 [16]. HMDAD collected MDA entries from 61 publications in microbiome studies based on 16s RNA sequencing. Each entry is an experimentally supported association between diverse microbes and non-infectious diseases with sufficient samples. HMDAD provides a benchmark source for developing prediction model [19].

**Fig. 3:**
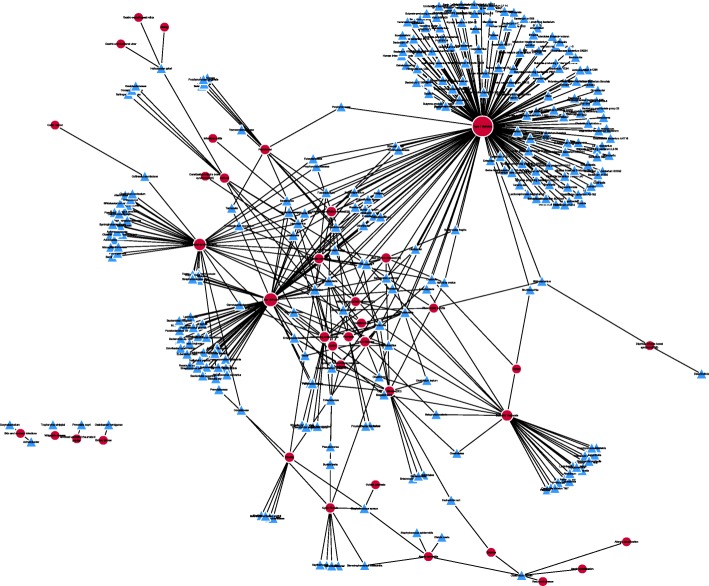
The Network of Microbe-Disease Associations. Blue triangles and red circles denote microbes and diseases respectively. Lines between nodes are the associations between them. The minimum, the median, the mean, and the maximum of microbe degrees are 1, 1, 1.54 and 11, while those of disease degrees are 1, 3, 11.54 and 167 respectively

Originally, there are 483 MDAs, including 292 microbes and 39 human diseases in the dataset. After removing the duplicate MDAs, which come from different experiments, Chen et al. [19] give 450 distinct MDAs among those microbes and diseases, and organizes them into a 292×39 association matrix. The corresponding MDA network is shown in Fig. 3.

### Parameter tuning

In this section, we investigated the influence of two important parameters in Formula 2, the standard derivation *σ* and the estimated rank *r*. First, we tuned it from the list {0.1, 0.2, 0.3, 0.4, 0.5, 0.6, 0.7, 0.8, 0.9, 1.0}. Since the maximum rank *r*_*max*_ of the underlying matrix is equal to min(*p*, *q*), we then tuned *r* from the ratio list of $\left\{\frac{1}{10},\frac{1}{9},\frac{1}{8},\frac{1}{7},\frac{1}{6},\frac{1}{5},\frac{1}{4},\frac{1}{3},\frac{1}{2},1\right\}$ w.r.t *r*_*max*_ and searched the best values on the 10 × 10 grid expanded by both *σ* and *r*.

**Fig. 4:**
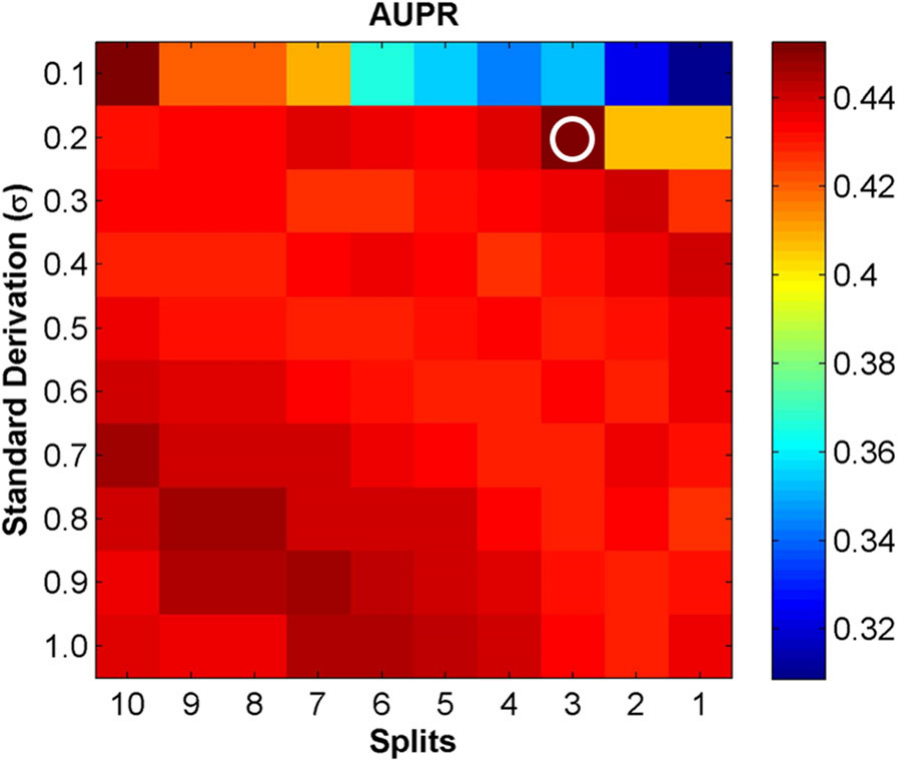
Illustration of Determining the Best Value Pair of (*σ*, *r*). The position w.r.t (*σ*^∗^, *r*^∗^) is highlighted by a white circle

Considering that AUPR is a better metric than AUC when the number of positive samples is significantly less than that of negative samples [24], we recorded the performance of BMCMDA for each pairwise value of (*σ*, *r*) under 5-CV in terms of AUPR (Fig. 4). When running BMCMDA, all the parameters (e.g. the number of iterations and the tolerance of stopping iteration) in SPG were set to their default values.

Finally, we picked up the pair of $\left({\sigma}^{\ast },{r}^{\ast}\right)=\left(0.2,\frac{1}{3}{r}_{max}\ \right)$, which achieves the highest one among 100 values of AUPR, as the best value of (*σ*, *r*), and further applied them in all the following experiments.

### Comparison with the state-of-the-art approach

**Fig. 5:**
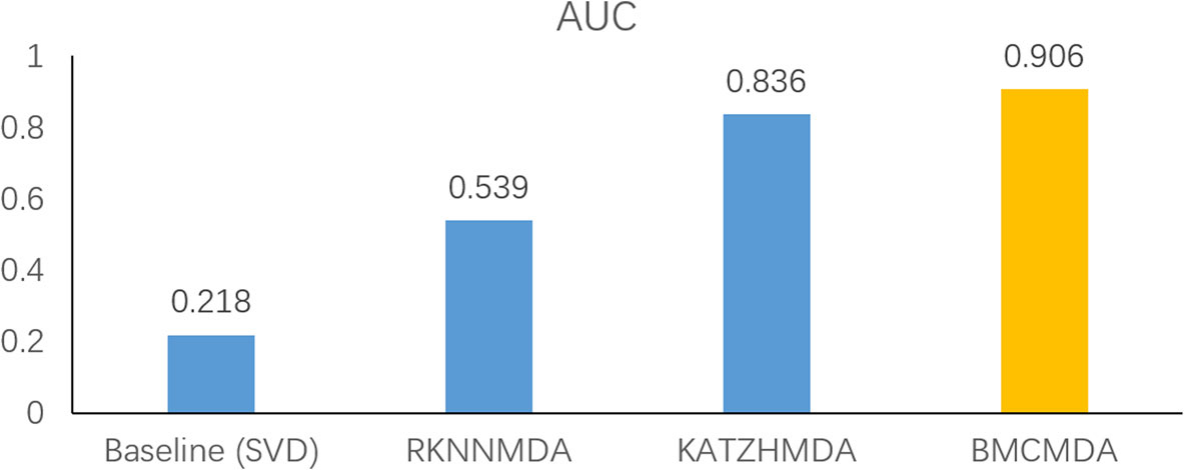
Comparison with state-of-the-art approaches

With the best pair (*σ*^∗^, *r*^∗^), we compared BMCMDA with three approaches, including one baseline approach and two state-of-the-art approaches, RKNNMDA [25] and KATZHMDA [19]. The baseline approach directly applies singular value decomposition (SVD) on the MDA adjacency matrix with missing entries and uses the product of two unitary matrices and the rectangle diagonal matrix to recover the missing values. RKNNMDA was originally designed for miRNA-disease associations [25]. It performs MDA prediction by directly applying a ranking-based KNN on the MDA prediction [19]. KATZHMDA also constructs a heterogeneous network, which consists of the known MDA network and two MDA-induced networks [19]. The first MDA-induced network indicates a microbe similarity network, while the second one accounts for a disease similarity network. Both of them are derived from the MDA network by Gaussian interaction profile kernel. By leveraging KATZ index to calculate similarities between microbe nodes and disease nodes in the heterogeneous network, KATZHMDA infers the potential association between a microbe node and a disease node if the value of their KATZ index is large. The comparison was performed with the exactly same dataset under LOOCV as mentioned in [19]. The results in Fig. 5. show that BMCMDA wins the best and outperforms those approaches significantly.

**Fig. 6:**
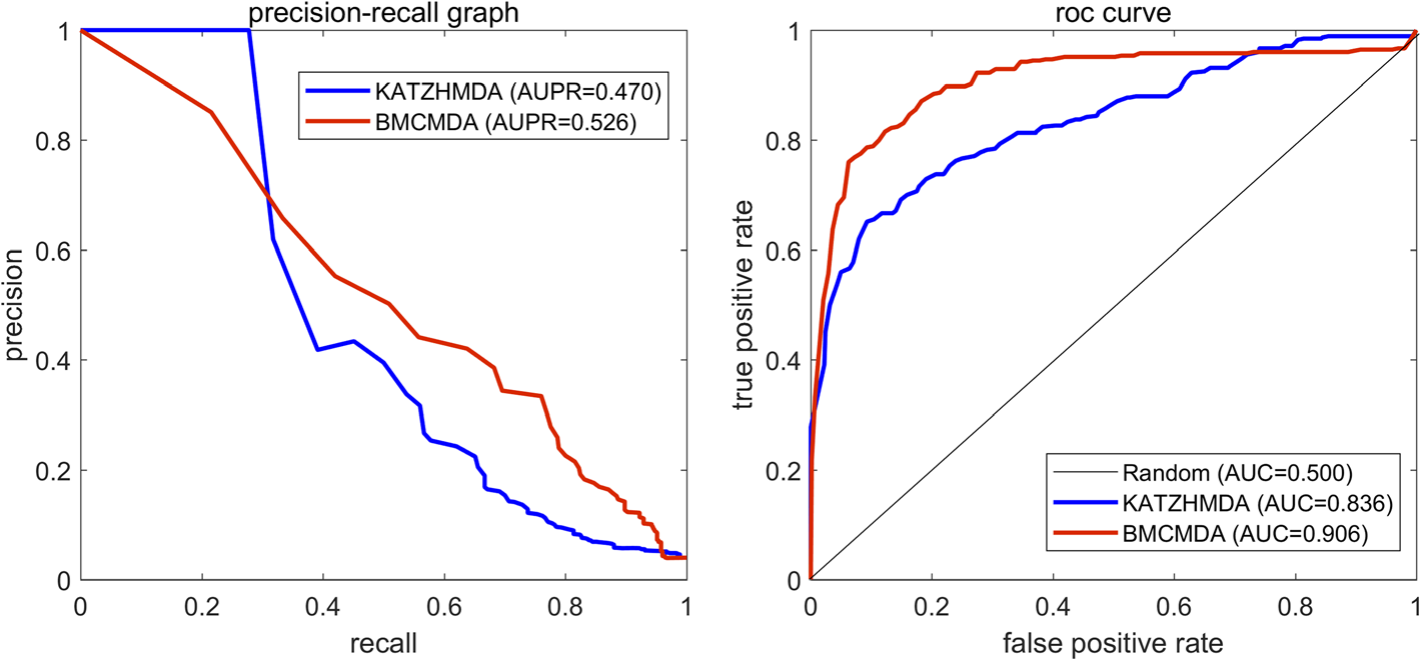
Comparison between BMCMDA and KATZHMDA in terms of ROC curve and PR curve

Furthermore, we selected the second best approach KATZHMDA to make a detailed comparison. Considering the fact that AUPR is a better metric than AUC when the number of positive samples is significantly less than that of negative samples [24], we measured the prediction by not only ROC curves but also PR curves. The results illustrated in Fig. 6 show that BMCMDA, compared with KATZHMDA, achieves a significant improvement of both ~ 7% increment in terms of AUC and ~ 5% increment in terms of AUPR.

## Conclusions

As the complement of biological experiments, computational methods have a potential to be a promising approach, which predicts MDA candidates rapidly among a plenty of microbe-disease pairs with the advantage of no limitation on microbe cultivation.

In this paper, we have modeled MDA prediction in a novel sight, which utilizes an underlying real-valued matrix to reflect the magnitude of MDAs and regards the binary MDA adjacent matrix as its incomplete and noisy observation. Upon this model, we have proposed a new approach based on Binary Matrix Completion (BMCMDA) to predict potential MDAs among a large scale of microbe-disease pairs. The comparison with other state-of-the-art approaches demonstrates the superiority of BMCMDA for predicting microbe-disease associations on a large scale and also validates that the assumption we adopted is reasonable. Obviously, BMCMDA can be directly applied to other similar forms of problems in bioinformatics, including the inference of the binary relationship between mono-partite objects (e.g. protein-protein interaction, drug-drug interaction [26, 27] and drug combination [28]) or that between bi-partite objects (e.g. drug-target interaction [29, 30], gene-disease association, RNA-disease association [31]).

In addition, we consider the possible improvement of BMCMDA. First, we may enhance the MDA prediction by integrating additional and independent microbe/disease similarities or features with BMCMDA. Secondly, as suggested in [31], we may generalize BMCMDA to be appropriate in more predicting scenarios, including the prediction of the associations between newly-found microbes (having no known MDA) and existing diseases, the prediction of the associations between existing microbes and newly-concerned diseases (having no known MDA), and the prediction of the associations between newly-found microbes and newly-concerned diseases.

## Abbreviations

AUC: The area under the receiver operating characteristic curve
AUPR: The area under precision-recall curve
BMCMDA: Binary Matrix Completion for predicting human Microbe-Disease Associations
CDF: Cumulative distribution function
CV: Cross-validation
HMDAD: Human Microbe-Disease Association Database
LOOCV: Leave-one-out cross-validation
MDA: Microbe-disease association
SPG: Spectral Projected Gradient
